# A Comprehensive Review of Multifunctional Nanozymes for Degradation and Detection of Organophosphorus Pesticides in the Environment

**DOI:** 10.3390/toxics12120926

**Published:** 2024-12-20

**Authors:** Jijia Liang, Zhongtian Dong, Ning Xu, Tao Chen, Jie Liang, Mingzhu Xia, Fenghe Wang

**Affiliations:** 1 School of Chemistry and Chemical Engineering, Nanjing University of Science and Technology, Nanjing 210094, China; ljj780208@163.com (J.L.); dzt97321@njust.edu.cn (Z.D.); ningxu202312@163.com (N.X.); 2 China Ordnance Equipment Group Automation Research Institute Co., Ltd., Mianyang 621000, China; ct718145100@126.com (T.C.); liangjie_1125@126.com (J.L.)

**Keywords:** organophosphorus pesticides, nanozymes, degradation, detection

## Abstract

Organophosphorus pesticides are the most extensively utilized agrichemicals in the world. They play a crucial role in regulating crop growth, immunizing against pests, and improving yields, while their unregulated residues exert serious detrimental effects on both the environment and human health. Many efforts have been made in the world to monitor organophosphorus pesticides and solve the issues caused by them. Nanozymes, as one kind of enzyme mimic that is artificially designed to simulate the function of natural enzymes, have aroused a lot of attention due to their unparalleled advantages. Nanozymes inherit both the unique properties of nanomaterials and catalytic functions, which could overcome the limitations inherent in natural enzymes and have great versatile and adaptable application prospects. This review presents a recent advancement in synthesizing multifunctional nanozymes with enzymatic-like activities by using various nanomaterials to degrade and detect organophosphorus pesticides. It mainly encompasses metal-based nanozymes, carbon-based nanozymes, metal–organic-framework-based nanozymes, and single-atom-based nanozymes. Additionally, this paper discusses the potential of nanozymes as novel functional environmental materials.

## 1. Introduction

**Figure 1:**
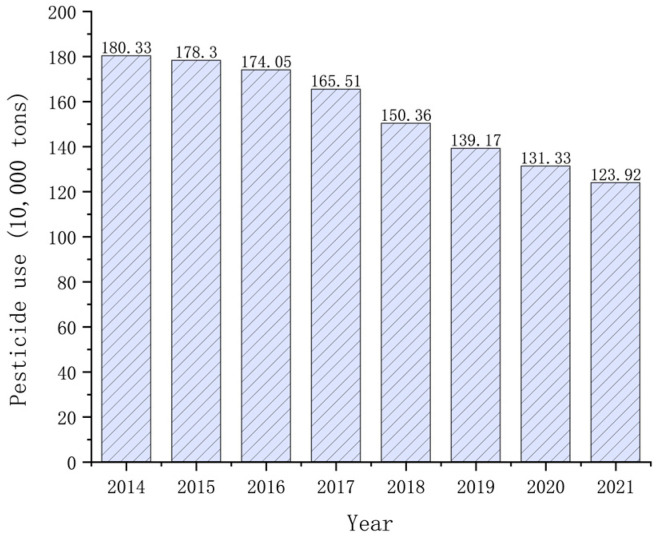
Annual pesticide usage data report from national statistics in China from 2014 to 2021 (https://data.stats.gov.cn/) (accessed on 4 December 2024).

Pesticides are powerful agents in managing crop cultivation, immunizing against pests and diseases, and regulating plant growth during agricultural production, which has significantly augmented crop yields and production [1]. Meanwhile, with the evolution of agricultural practices and more demand for agricultural products, the utilization of pesticides has also increased significantly. From 2000 to 2015, the usage of pesticides has witnessed a substantial rise of 40% [2]. According to national statistics in China (illustrated in Figure 1), although there was a minor decline in pesticide usage from 2014 to 2021, it remained at a notably elevated level (https://data.stats.gov.cn/) (accessed on 4 December 2024). The global average pesticide application escalated from 2.28 kg/ha in 2005 to 2.69 kg/ha in 2019 (http://www.fao.org/home/en/) (accessed on 4 December 2024). Pesticide usage in the United States has surpassed the global average, increasing from 2.89 kg/ha in 2005 to 3.70 kg/ha in 2019 [3]. China, which holds 7% of the world’s arable land, stands as one of the largest consumers of pesticides, accounting for 35% of global pesticide and fertilizer usage. Because of their effectiveness in pest and disease control, chemical pesticides have played a pivotal role in enhancing crop yields [4]. Therefore, it is anticipated that the widespread development and deployment of pesticides will continue to expand in the future [5]. However, despite the advantages, excessive OP usage and inappropriate residue disposal can lead to pest resistance [6], environmental pollution [7,8], and harm to human health [9].

**Figure 2:**
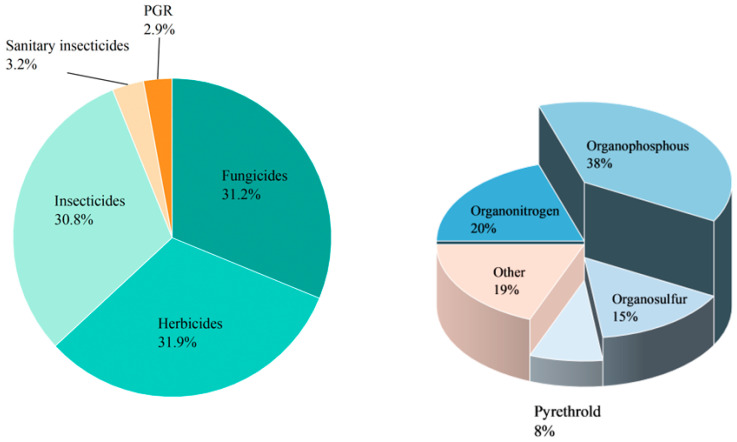
Classification and application of various pesticides in Chinese agriculture (PGR—plant growth regulators, also known as plant regulators, refer to pesticides that regulate the growth and development of plants; sanitary insecticides refer to pests that are mainly used in the field of public health to control vector organisms and affect the lives of people).

Pesticides are diverse and can be classified by chemical categories, functional groups, modes of action, and toxicity [10]. Based on their chemical composition, pesticides can be broadly divided into inorganic and organic types. Inorganic pesticides contain elements such as sulfur and copper, and common inorganic insecticides include copper sulfate, ferrous sulfate, copper, lime, and sulfur [11]. Organic pesticides contain elements such as fluorine, chlorine, sulfur, phosphorus, oxygen, and carbon. Figure 2 shows the classification and application of various pesticides in China. Among them, organophosphorus pesticides are the most commonly used pesticides, accounting for more than 34% of total pesticide usage. They were introduced in the 1950s and are used for fruits, vegetables, and other crops [12]. Organophosphates originate from phosphoric acid, which contains mainly phosphorus, carbon, and oxygen atoms. They are widely disseminated in many countries to replace organochlorines due to their easier degradation in the environment and shorter half-lives. It is anticipated that organophosphorus pesticides, which comprise approximately 40% of the global market, will continue to hold a prominent position in the coming years. Most organophosphorus pesticides produced in China are insecticides, such as malathion, parathion, omethoate, and dichlorvos. The molecular structures of organophosphorus pesticides are diverse, typically containing bonds such as P=O, P=S, C−P, C−N−P, C−O−P, and C−S−P. Many are poorly soluble or marginally soluble in water but readily dissolve in organic solvents. Depending on the double-bond substitution element, the molecular structure of organophosphorus pesticides can generally be divided into two categories: organophosphorus pesticides with a double-bond substituent of =O, such as dichlorvos and omethoate, and organophosphorus pesticides with a double-bond substituent of =S, including malathion and parathion.

Although organophosphorus pesticides play a great role in the improvement of crop yield and quality, pest control, and plant growth regulation, their excessive usage and inappropriate residue disposal can pose significant threats to human health, potentially causing various functional disorders. The toxicity of organophosphorus pesticides varies, with most being highly toxic, while some exhibit lower toxicity. The toxicity of common organophosphorus pesticides is shown in Table 1. They form phosphorylated cholinesterase covalently in the body, inhibiting cholinesterase activity, preventing the breakdown of acetylcholine [13], leading to a massive accumulation of acetylcholine at synapses, causing depolarization of the postsynaptic membrane, preventing neural conduction, and causing neurological disorders [14]. Organophosphorus pesticides can enter the human body via multiple pathways, including the digestive tract, respiratory system, skin, or mucous membranes, which would pose a significant threat to human health. Mild cases may cause acute poisoning, resulting in symptoms such as shortness of breath, arrhythmia, hypoxia, and coma. Occupational pesticide poisoning can occur due to skin contamination [15]. Long-term organophosphorus pesticides accumulation can lead to internal organs, such as the liver and heart, damage.

By reviewing the literature on organophosphorus pesticides from 2000 to 2022, we summarized the detection of organophosphorus pesticides in various waterbodies (Table 2). As shown in the table, the main organophosphorus pesticides found in Chinese water bodies are omethoate and dichlorvos. The concentration of chlorpyrifos in Malaysian river water was as high as 5057 ng L^−1^, and the concentration of triazophos in the Nile River in Egypt was as high as 2600 ng L^−1^. Different crop types, farming methods, and development levels in different countries could cause various organophosphorus pesticides usage, which will lead to different types and concentrations in waterbodies across the world. The diversity and complexity of organophosphorus pesticides residues will pose significant challenges for detection efforts.

Because of their exceptional specificity and remarkable catalytic performance, natural enzymes are extensively utilized in bioassays. However, their catalytic function depends on the secondary structure of proteins, which can easily denature under high temperatures, strong acids and bases, or in the presence of organic solvents. This limitation greatly restricts their applications. Recently, some nanomaterials have demonstrated catalytic activities like those natural enzymes, with reactions conforming to the fundamental kinetic principles and mechanisms of enzymes. Nanozymes, a novel type of artificial enzyme, are different from natural enzymes, traditional small-molecule mimics, and chemical catalysts. Combining the properties of nanomaterials and enzymes, nanozymes represent a promising enzyme substitute with unique physicochemical properties and biological functions, including designability, ease of large-scale production, cost-effectiveness, modifiability, high stability, and the ability to regulate catalytic activity and types through external stimuli [24,25]. Due to the significant advancements in nanotechnology and the unique properties of nanomaterials, they have played notable roles in different fields, making substantial progress and attracting great attention around the world [26]. On one hand, nanozymes exhibit high enzymatic-like catalytic activity to modulate biochemical reactions; on the other hand, they inherit the properties of nanomaterials and have the ability to overcome the shortcomings of natural enzymes, which have great potential in serving as a multifunctional application platform [27]. To date, with the identification of new features, various nanozymes have been successfully applied in diverse fields, including biosensing [28], cancer treatment [29], food safety, sterilization [30,31], and environmental purification [32].

## 2. Migration and Transformation of Organophosphorus Pesticides in the Environment

**Figure 3:**
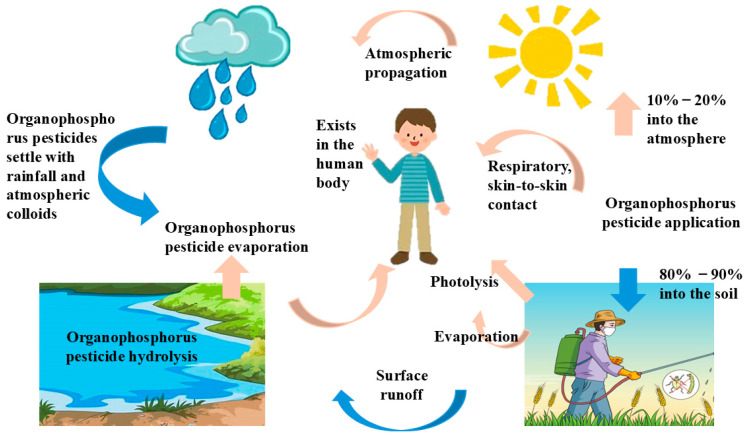
Migration and transformation of organophosphorus pesticides in the environment and food chain.

When organophosphorus pesticides are applied, 10~20% enter the air and are absorbed by plants, while approximately 80~90% diffuse into other environmental media, such as air, water, and soil, rather than directly affecting target organisms. As shown in Figure 3, sprayed organophosphorus pesticides can enter waterbodies through drift, runoff, leaching, and subsurface drainage, where they are absorbed by algae, fish, and other organisms. Some of these organophosphorus pesticides enter the atmosphere via volatilization, photodegradation, and atmospheric transport, subsequently settling back to the earth with rainfall and atmospheric particulates. Others adhere to soil, move within it, or undergo microbial degradation, forming bound residues in soil and sediments, potentially disrupting and damaging ecosystems. Simultaneously, organisms come into contact with organophosphorus pesticides through inhalation, ingestion, and dermal absorption from various sources, including air, water, soil, and other organisms.

Given the drawbacks of organophosphorus pesticides, they have large usage and complex composition. For the damage, they are nonbiodegradable and have high toxicity, significant risks, and widespread harm. Meanwhile, they are difficult to detect and are easy to migrate and transform in the environment. They can also accumulate in the food chain, which poses a severe threat to the environment and human health. It is therefore essential to monitor organophosphorus pesticides in the environment, drinking water, food, and biological fluids, and it is of great importance to conduct research on their degradation and detection, which holds profound environmental significance.

## 3. Bibliometric Analysis of Research on Degradation and Detection of Organophosphorus Pesticides

**Figure 4:**
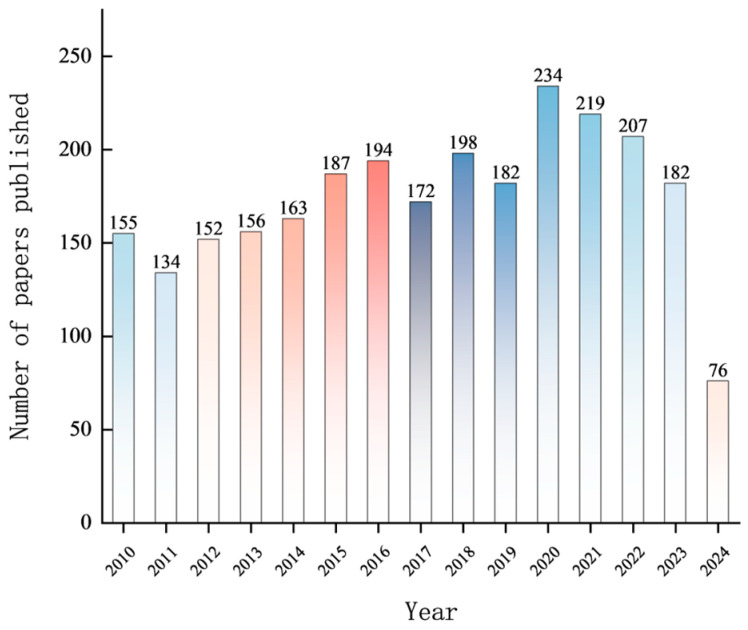
Annual publication volume of studies related to the degradation and detection of organophosphorus pesticides in the WoS database.

A thematic search was conducted in the Web of Science (WoS) core database by using the keywords “organophosphorus pesticides” and “degradation or determination”. The document type was set to “article”, the language to “English”, and the time range to January 2010 to December 2024. After refinement, a total of 2611 relevant documents were retrieved. Figure 4 illustrates the annual publication volume of studies related to the degradation and detection of organophosphorus pesticides in the WoS database. As shown in Figure 4, with a slight decline over the past three years, the overall trend of publications on organophosphorus pesticides degradation and detection in the WoS database is increasing. The highest number of publications was in 2020, with 234 articles, indicating continued interest in this research area. The research in this area continues to gain popularity. In terms of publication volume, China leads with 1034 articles, followed by Iran (358 articles), India (219 articles), and the United States (129 articles).

A thematic search was conducted in the Web of Science (WoS) core database using the keywords “organophosphorus pesticides” and “nanozyme”. The document type was set to “article”, the language is “English”, and after refinement, a total of 99 relevant documents were retrieved. By categorizing all extracted keywords with CiteSpace 6.4.R1 software, the intrinsic logical relationships were characterized. Besides the current thematic keywords ‘nanoparticles’ and ‘organophosphorus pesticides, the most frequent term is ‘colorimetric detection’ (16 times), indicating that colorimetric detection is a crucial method for detecting organophosphorus pesticides using nanozymes. Acetylcholinesterase appeared 12 times, highlighting its significance in the inhibition of acetylcholinesterase by organophosphorus pesticides during the degradation and detection process. Metal–organic frameworks appeared 11 times, suggesting that metal–organic frameworks, as emerging nanozymes, are receiving considerable attention in the field of organophosphorus pesticides.

## 4. Phosphoester Nanozymes and Their Research Progress

**Figure 5:**
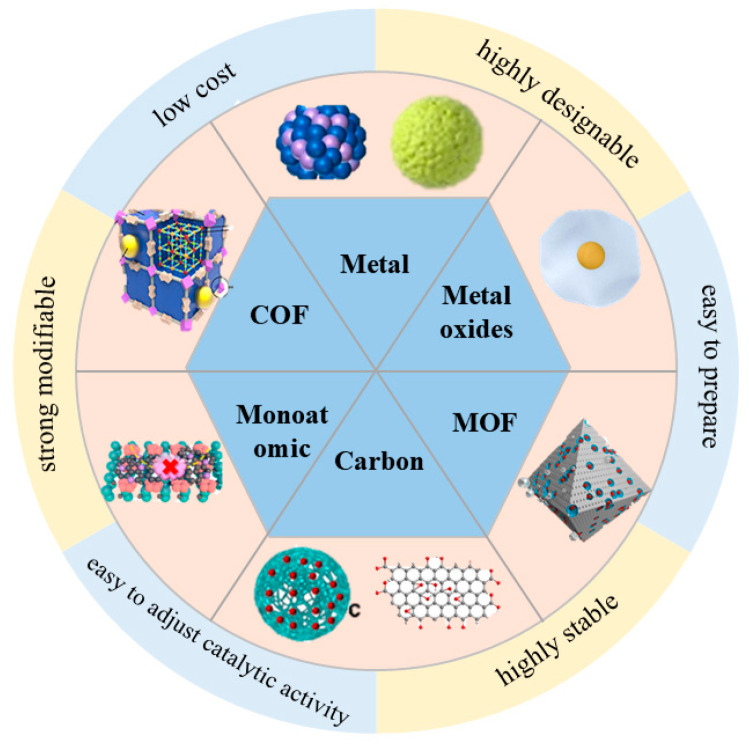
Properties and classification of nanozymes used for the degradation and detection of organophosphorus pesticides.

In this review, nanozymes used for the degradation and detection of organophosphorus pesticides are divided into six categories depending on the composition and structure: metal-based nanozymes, metal oxide nanozymes, metal–organic framework (MOF)-based nanozymes, single-atom nanozymes, carbon-based nanozymes, and covalent organic framework (COF)-based nanozymes. Properties and classification of nanozymes used for the degradation and detection of organophosphorus pesticides are clearly shown in Figure 5.

### 4.1. Metal-Based Nanozymes

As shown in Table 3, there are several examples of metal-based nanozymes used for the degradation and detection of organophosphorus pesticides.

Most metal-based nanozymes exhibit superior enzymatic activity due to their excellent conductivity, which promotes electron transfer between the enzyme and the electrode surface. Currently, lanthanide ions (Eu^3+^, Ce^3+^, Er^3+^, Sm^3+^, Tb^3+^, La^3+^, Nd^3+^) and transition metal ions (Zr^4+^, Ni^2+^, Cu^2+^, Zn^2+^, and Co^2+^) are commonly used for the hydrolysis of phosphate esters. Copper-based nanomaterials, known for their excellent biocompatibility and unique physicochemical properties, are extensively employed in the synthesis of nanozymes [44,45]. Copper(II) can hydrolyze certain organophosphorus pesticides via bidentate coordination involving nitrogen within the ring structure and sulfur in the phosphate side chain [46]. Thus, copper-based nanozymes show great potential in the hydrolysis and recognition of organophosphorus pesticides. Song et al. [33] developed a novel sensor array based on four copper-based nanozymes with laccase-like activity, is capable of detecting six organophosphorus pesticides (Iso, Mal, Pho, Gly, Glu, Fen). The results showed that the sensor array could selectively respond to different organophosphorus pesticides and achieved selective recognition and individual identification. The sensor array has differentiated fingerprints of various concentrations of organophosphorus pesticides (1, 5, 20, 50, 100 μg mL^−1^), achieving complete separation of each concentration, thereby showcasing its exceptional differentiation capability over a wide range [33]. The π–π interactions and electrostatic interactions significantly enhance electron transfer within nanozymes, thereby boosting their stability and catalytic activity. Niu et al. [35] synthesized sulfonated cobalt phthalocyanine nanozymes (CoPcNS) using a straightforward one-pot hydrothermal method to fabricate an electrochemical sensor based on acetylcholinesterase (AChE). The linear range was 10~2000 μg L^−1^, and the detection limit for parathion was 1.1 μg L^−1^ [35]. Phosphotungstic acid containing iron, as a member of the polyoxometalates family, exhibits remarkable peroxidase-like activity under neutral conditions. Zhu et al. [36] discovered that this iron-containing phosphotungstic acid (Fe-PTs) maintains exceptional peroxidase-like activity in neutral conditions. By integrating Fe-PTs with AChE and choline oxidase (ChOx), they developed a multi-enzyme cascade system for the detection of organophosphorus pesticides. The detection range for parathion was 1~500 ng mL^−1^, with a detection limit of 0.28 ng mL^−1^. Jiang et al. [42] discovered that platinum–nickel nanoparticles (Pt-Ni NPs) nanozymes possess excellent oxidase-like activity. These nanozymes induce the oxidation of colorless 3,3′,5,5′-tetramethylbiphenyl (TMB) to form blue-colored ox-TMB. Based on this principle, they developed a colorimetric/photothermal dual-mode probe to detect organophosphorus pesticides, using chlorpyrifos as a model compound. The detection limits for the colorimetric and photothermal modes were 1.2 ng mL^−1^ and 1.66 ng mL^−1^, with linear ranges of 0.2~2.5 μg mL^−1^ and 0.005~3.0 μg mL^−1^, respectively [42]. Weerathunge et al. [41] utilized tyrosine-capped silver nanoparticles (AgNP) known as Ag-NanoZyme and coupled them with specific aptamers for chlorpyrifos to construct a colorimetric aptasensor. When the sensor probe makes contact with chlorpyrifos, the colorless substrate TMB converts blue, with the concentration of chlorpyrifos added and the color intensity increasing. The detection limit and quantification limit were 11.3 ppm and 34.1 ppm, respectively, with a linear range of 35~210 ppm [41].

Currently, numerous research teams enhance the stability and catalytic activity of nanozymes by altering their surface chemistry, such as through surface modification and doping techniques, to introduce additional active sites within the nanostructure. Zou’s group utilized Fe-doped Bi metal–organic frameworks (FeBi MOFs) to synthesize Fe/C/Bi_2_O_3_ nanozymes. By leveraging Fe as a strong catalytically active site with the synergistic effect between the two metals, they enhanced the peroxidase-like activity of the nanozymes. Further, by combining them with AChE and choline ChOx to form a tri-enzyme cascade system, they achieved colorimetric sensing for dichlorvos within the linear range of 10~100 μg L^−1^, and with a detection limit of 0.6 μg L^−1^ [37]. Additionally, Ji et al. [38] established a multi-enzyme-mediated electrochemical biosensor (MRMEC) by immobilizing the peroxidase-like Fe_3_O_4_@Au-Pt nanozymes onto graphene nanocomplexes (GN-Au NPs) modified glassy carbon electrodes (GCE). In this system, Fe_3_O_4_@Au-Pt and GN-Au NPs serve as the catalyst and signal amplifier, respectively. This sensing platform is suitable for detecting the residue of ethephon (ETH) in fruits and vegetables, with a detection limit of 2.01 nmol L^−1^. Au nanoparticles, known for their unique optical and electronic properties, are often chosen as materials for biosensor fabrication. Yan’s group synthesized Bi_0.01_Au_1_ nanozymes by introducing Bi atoms into pure gold aerogels, significantly enhancing their peroxidase-like activity. They established a colorimetric sensing platform for detecting organic phosphorus pesticide residues, using paraoxon–ethyl as a model compound. Within the linear range of 0.8~500 ng mL^−1^, the detection limit was 0.41 ng mL^−1^ [34]. Lei et al. [40] doped trace amounts of Bi into core–shell Pd@Pt mesoporous nanospheres, which possess high electron transfer rates, resulting in the synthesis of a dual-doped core–shell Pd@Pt mesoporous nanosphere (Pd@PtBi_2_) with enhanced peroxidase-like activity. The introduction of trace Bi significantly boosted the peroxidase-like activity of the mesoporous nanospheres. They further established a biosensor based on AChE, which achieved a detection limit as low as 0.06 ng mL^−1^ for trichlorfon within the range of 0.1~100 ng mL^−1^, observing a good linear relationship between the absorbance and trichlorfon concentration. Apart from metal atoms, nitrogen (N) atoms are also commonly used as dopants. Another research team combined ultrathin two-dimensional graphitic carbon nitride (g-C_3_N_4_) nanosheets with PtPd nanoparticles. Shen et al. [43] leveraged the oxidase-like activity and fluorescence properties of the PtPd nanoparticles to successfully establish a colorimetric–ratiofluorescence dual-mode detection method. Under visual colorimetric mode, the linear range for detecting trich was 0.28~50.0 ng mL^−1^, with a detection limit of 0.083 ng mL^−1^. In the ratiometric fluorescence mode, the linear range was 0.11~50.00 ng mL^−1^, with a detection limit as low as 0.033 ng mL^−1^ [43].

Most detection methods utilizing nanozymes predominantly depend on the singular peroxidase-like activity [47]. However, numerous peroxidase-like nanozymes also exhibit oxidase-like activity, such as Co_3_O_4_ [48], MnO_2_ [49], Pt [50], and Au [51]. If these nanozymes possess both activities, they can generate higher responses and mitigate the interference of oxygen on the peroxidase-like activity detection results. For instance, Liang et al. [39] employed polyvinyl alcohol as a stabilizer and reduced Ir^3+^ precursors with ethanol to successfully prepare well-dispersed Ir nanoparticles (Ir NPs). Under identical conditions, these Ir nanoparticles demonstrated both peroxidase-like and oxidase-like activities, with distinct catalytic mechanisms. Leveraging the high sensitivity and selectivity of malathion inhibition on the two enzyme activities of Ir NPs, a colorimetric detection platform without enzymes was established. As the quantity of malathion increased, the blue color gradually faded, with corresponding absorbance peaks progressively diminishing. Within the range of 0.1~5.0 μM, there was a strong linear relationship between absorbance and malathion concentration, with an estimated detection limit of 6 nM.

### 4.2. Metal Oxide Nanozymes

Table 4 shows the application and performance of metal oxide nanozymes in the degradation and detection of organophosphorus pesticides.

In comparison to conventional bulk metal oxide materials, nano-metal oxide materials exhibit superior catalytic performance. Metal elements such as Ce, Gd, La, Ti, Zr, and Mn are frequently used to construct oxide-type phosphoester nanozymes. These oxide nanozymes with different compositions display distinct catalytic activities and selectivities. Furthermore, novel nano-metal oxide materials like cerium oxide–titanium oxide (CeO_2_-TiO_2_) and zirconium oxide–cerium oxide (ZrO_2_-CeO_2_) nanomaterials have also been found to possess excellent phosphoester catalytic properties. This indicates a broad prospect for nano-metal oxide materials in the study of phosphoester nanozymes. CuO nanozymes with peroxidase-like activity can oxidize o-dianisidine in the presence of hydrogen peroxide and acetylthiocholine. Arsawiset et al. [52] designed an oxidized copper nanozyme (CuONPs) paper-based analytical device and modified it with silicon oxide nanoparticles (SiONPs). This CuO nanozyme exhibits stronger catalytic activity than peroxidase. Utilizing the fact that an organophosphorus pesticide like malathion is converted by cytochrome P450 enzymes to malaoxon, which inhibits the activity of AChE, with o-dianisidine as a chromogenic substrate, the rapid detection of organophosphorus pesticides in fruits and vegetables is possible by monitoring the color change to brown. This nanozyme-paper-based analytical device offers a linear range of 0.1~5 mg L^−1^, with a low detection limit for malathion (0.08 mg L^−1^) and a short analysis time (approximately 10 min). Liang et al. [47] synthesized GeO_2_ nanozymes with peroxidase-like activity using a hydrothermal synthesis method, utilizing TMB as a substrate, and established a colorimetric detection strategy combined with AChE. This strategy achieves a detection limit of 14 fM for parathion, with a linear range of 0.1~50 pM. Jing et al. [53] designed a colorimetric sensor array without natural enzymes by using the oxidase-like activity of Ag_2_O. This array can simultaneously detect six organophosphorus pesticides: fenitrothion, chlorpyrifos, omethoate, triazophos, methyl parathion, and trichlorfon. Based on the inhibition of Ag_2_O nanospheres by the presence of organophosphorus pesticides, a color pattern was generated for each sample. By integrating with a smartphone, rapid visual detection of organophosphorus pesticides is facilitated. The sensor array could successfully identify organophosphorus pesticides down to a concentration of 10 ng mL^−1^. Huang et al. [54] used degradable manganese oxide nanowires (γ-MnOOH NWs) as degradable nanozymes with oxidase-like activity. TMB was used as a chromogenic indicator with AChE, then, a simple colorimetric paper sensor for organophosphorus pesticides detection was established, allowing for rapid readout of AChE activity or organophosphorus pesticides quantity from color signals. The linear range for omethoate is 5~50 ng mL^−1^ with a LOD of 0.35 ng mL^−1^, and for dichlorvos, the linear range is 1~10 ng mL^−1^ with a LOD of 0.14 ng mL^−1^. Furthermore, other researchers synthesized ultra-thin FeOOH-coated MnO_2_ to reduce the agglomeration of manganese oxide. Based on the MO@FHO nanozyme, they constructed a photoelectrochemical (PEC)/colorimetric method for detecting malathion. In the PEC mode, the detection limit for malathion within the range of 0.0001~0.5 μmol L^−1^, and the LOD was 0.17 nmol L^−1^. In the colorimetric mode, the detection limit for malathion within the range of 0.001 to 50 μmol L^−1^, and the LOD was 0.8 nmol L^−1^ [55].

Researchers have proposed using substrate synergy to enhance the hydrolytic activity of nanoparticles by anchoring metal-active centers on substrates with high adsorption capacity. Gai et al. [56] designed a novel CeO_2_@NC nanozyme in 2023 by embedding CeO_2_ nanoparticles into a nitrogen-doped carbon material substrate. This accelerates electron transfer between the metal-active center and the substrate interface, thereby enhancing its phosphatase-like activity. This nanozyme can rapidly dephosphorylate under mild conditions, extending beyond high-temperature and high-concentration regimes. With a Ce(SO_4_)_2_ concentration of 3.5 mM, optimal catalytic performance is observed, achieving rapid hydrolysis of parathion within 10 min at low temperatures (37 °C) and low dosages (0.5 mg mL^−1^). A simple colorimetric detection method was also established by using this nanozyme, which specifically detected parathion in the range of 3.0~100.0 μM through dephosphorylation reactions. Metal element doping is another crucial pathway to regulate the activity of nanozymes. Zhao et al. [57] synthesized a hybrid nanozyme (Au-pCeO_2_) with phosphatase-like activity by modifying porous CeO_2_ (pCeO_2_) with gold nanoparticles (AuNPs). The modified nanozyme exhibits enhanced catalytic activity. A colorimetric sensor for detecting methyl parathion (MP) was developed; MP was detected and analyzed by using UV–visible spectrophotometry and a smartphone. The detection limit was 0.5 μM, with a detection range of 5~200 μM. Au nanoclusters (AuNCs) are extensively utilized in biosensing due to their advantages of small size and large Stokes shift. Zhang et al. [58] combined AuNCs with molecularly imprinted polymers (MIPs), polydopamine (PDA), and hollow CeO_2_ nanospheres to synthesize a novel core–shell fluorescent probe (CeO_2_@PDA@AuNCs-MIPs). Ce(IV)/Ce(III) can act as a hydrolytic active center, attacking the P−O bond of methyl parathion (MP) to produce p-nitrophenol (p-NP), which quenches the fluorescence of the polymer. Ce(III) can also serve as a fluorescence signal amplifier. Based on this nanozyme, the MP fluorescence detection method exhibits excellent performance with a linear range of 0.45~125 nM and a detection limit of 0.15 nM.

Utilizing defect engineering at the interface, the creation of vacancies significantly enhances the oxidase-like activity of the nanozymes. CeO_2_ nanozymes have been extensively studied for their phosphatase-like activity in hydrolysis processes. Miura-Stempel’s group used trivalent M^3+^ dopants to modulate the catalytic activity of nanozymes by adjusting the relative concentrations of Ce^3+^ and oxygen vacancies. Their research is on the impact of trivalent dopants, such as Y^3+^, Cr^3+^, In^3+^, and Gd^3+^; the hydrolysis of dimethyl-p-nitrophenyl phosphate (DMNP) revealed that In-CeO_2_ exhibited a hydrolysis rate approximately 1.5 times higher than CeO_2_. This enhancement is attributed to its defect surface containing Ce^4+^, the formation of oxygen vacancies by In^3+^/Ce^3+^, and the presence of −OH coordinated with adsorbed substrates near Ce^4+^ [59]. Zeng’s group designed highly oxidized Au@MnO_2−X_ nanozymes featuring a core–shell nanostructure. The core–shell architecture and ultra-thin morphology significantly increase surface defects, and the high concentration of oxygen vacancies enhances their extraordinary oxidase-like activity. Based on this, they successfully constructed an electrochemical sensor for detecting organophosphorus pesticides using homogeneous electrochemical chemistry (HEC). The detection limit for the sensor is 0.039 ng mL^−1^, with a linear range of 0.01~50.0 ng mL^−1^, capable of linearly discriminating between five types of organophosphorus pesticides: Ethion, Omethoate, Diazinon, Chlorpyrifos methyl, and Dipterex [60].

### 4.3. MOF-Based Nanozymes

From Table 5, we can clearly see the performance of several MOF-based nanozymes in the degradation and detection of organophosphorus pesticides.

#### 4.3.1. Pure MOFs Materials

MOFs are porous hybrid polymers assembled through modular organic ligands and metal ions (or metal clusters). The wide array of metal ions and organic ligands can be combined through various coordination methods, providing MOFs with a high degree of structural and functional tunability. These materials exhibit well-defined secondary structures, metal-active sites, and functional ligands, forming specific frameworks that enable precise regulation of catalytic activities and support comprehensive mechanistic studies. The ability to fine-tune the composition and arrangement of these components allows for the customization of MOFs to meet specific catalytic requirements, thereby enhancing their effectiveness in diverse applications. Additionally, MOFs can be synthesized under mild and controllable conditions, with metal ions or organic bonds readily amenable to design and modification. The inherent connectivity and topological structure of MOFs remain unchanged due to their specific pore configurations. The enzyme-like catalytic capabilities of MOFs arise from two primary factors. Firstly, the nodes containing redox-active metals (such as Ce, Co, Fe, Cu, etc.) provide enzyme-like catalytic activities. Secondly, certain organic ligands within MOFs function as electron mediators, accepting electrons from one substrate and transferring them to another, exhibiting catalytic activities similar to those of natural enzymes. This dual mechanism not only enhances the catalytic efficiency but also allows for precise control over the catalytic processes. The integration of redox-active metal nodes and electron-transfer ligands contributes to the multifunctional and versatile catalytic properties of MOFs, making them promising candidates for various catalytic applications [78].

Research has shown that the utilization of synergistic effects between different valence states of Ce ions can significantly enhance the hydrolytic activity of Ce-based MOFs. Yuan et al. [61] designed a series of ultra-small Ce-MOF nanozymes using 2-methylimidazole as the organic ligand. They synthesized Ce-MOF(aq) at room temperature in aqueous solutions, Ce-MOF(DMF) at elevated temperatures using DMF as the solvent, and MOF(MeOH) at room temperature using methanol as the solvent. Results indicated that under the same Ce concentration, the catalytic activity of Ce-MOF(aq) was 3.7 times and 14.9 times higher than that of Ce-MOF(DMF) and Ce-MOF(MeOH), respectively. In CHES buffer (pH 9.0), Ce-MOF(aq) achieved an 80% hydrolysis rate of para-nitrophenyl phosphate (p-NPP) within 5 min and a 98.5% hydrolysis rate within 25 min. Additionally, it realized the hydrolysis of environmental pollutants or organophosphorus pesticides Bis(4-nitrophenyl) phosphate (BNPP), methyl parathion, and DMNP, with hydrolysis rates of 76.94%, 34.1%, and 82.56%, respectively. These findings indicate that Ce-MOF nanozymes exhibit excellent phosphatase-like hydrolytic activities [61]. Moreover, Xiao et al. [62] successfully synthesized Cu_4_Co_6_ ZIF nanozymes with high peroxidase-like activity. These nanozymes are capable of catalyzing the generation of hydroxyl radicals (•OH) from hydrogen peroxide (H_2_O_2_), leading to the oxidation of TMB and the production of a strong blue color. Specifically, the degree of blue color decreases as the concentration of organophosphorus pesticides increases, enabling a colorimetric detection method without AChE to directly detect organophosphorus pesticides, which provided a visual indication of the presence and concentration of the target analyte. The detection range for pirimiphos-methyl is 6 × 10^−4^~0.03 μM, with a detection limit of 0.151 nM [62]. Du et al. [63] utilized V^3+^ with peroxidase-like activity, synthesizing vanadium-based MOFs (MIL-88B(V)) through solvothermal methods. Organophosphorus pesticides can be fixed on this MOF material via V−O−P bonds, reducing its catalytic activity. Based on this, a smartphone colorimetric sensing platform was developed for visual detection of ethion, parathion, dichlorvos, and paraoxon, with detection limits of 0.018, 0.01, 0.02, and 0.027 μg mL^–1^ [63].

#### 4.3.2. Composite MOFs Materials

Previous studies to enhance the catalytic performance of MOFs have often involved surface modifications and doping. By leveraging the excellent synergistic electronic transfer effects between different metals, atomic or non-metallic dopants can be introduced into MOFs to enhance their catalytic performance. For instance, Luo et al. [64] incorporated manganese ions into a typical iron-based MOF (Fe-MIL(53)) through a one-pot hydrothermal reaction, producing a dual-metal MOF (Mn/Fe-MIL(53)) with superior catalytic properties. This enhanced its oxidase-like activity significantly. The colorimetric detection system established for this MOF achieved the detection limits of 2.8 nM and 0.95 nM for methyl parathion and chlorpyrifos, respectively, with linear ranges of 10~120 nM and 5~50 nM [64]. Feng et al. [65] used a one-pot thermal method to dope manganese ions into Zeolitic Imidazolate Framework (ZIF-8), resulting in Mn-ZIF-8 nanozymes with higher peroxidase-like activity due to increased active sites. This allowed for the specific recognition of organophosphorus pesticides, with a detection limit of 54 pM for chlorpyrifos within the range of 0.1~20 nM [65]. Xu et al. [66] introduced nitro (−NO_2_) and amino (−NH_2_) functional groups with opposite electronic modulation capabilities into a typical iron-based MOF (MIL-101(Fe)), creating NO_2_-MIL-101 and NH_2_-MIL-101, respectively. By controlling atomically dispersed metal sites, the peroxidase-like activity was regulated. Introducing the electron-withdrawing −NO_2_ group enhanced MIL-101′s peroxidase-like activity, whereas the electron-donating −NH_2_ group had the opposite effect. A colorimetric biosensor was then developed based on NO_2_-MIL-101 for sensitive detection of organophosphorus pesticides, achieving a detection limit of 1 ng mL^−1^ for methyl parathion within the range of 8~800 ng mL^−1^ [66]. Shen et al. [67] synthesized hydrophobic Pt@ZIF-8@TMS nanozymes by incorporating tetraethyl orthosilicate (TMS) into ZIF-8. Based on this, a colorimetric biosensor was developed, achieving a detection limit of 0.7 ng mL^−1^ for malathion within the range of 0~500 ng mL^−1^ [67]. Loading onto the surface of MOF materials is also a common approach to enhance catalytic activity. Yi et al. [68] synthesized an Fe-based MOF (MIL-888-NH_2_) and loaded platinum nanoparticles (Pt NPs) onto its surface to form Pt NPs/Fe-MOF composite nanozymes. Based on this nanozyme, a simple colorimetric detection method was constructed, with a detection range for dichlorvos of 0.01~10.0 ng mL^−1^ and a detection limit of 2.9 pg mL^−1^. Further experiments showed that this method could also detect other organophosphorus pesticides, including trichlorfon and chlorpyrifos. Jin et al. [69] used qualitative filter paper (FP) as a substrate and grew Ce/Zr-MOF via solvothermal methods on FP, obtaining Ce/Zr-MOF@FP. This sensor exhibited a detection limit of 0.32 ng mL^−1^ within the concentration range of 0.5~500 ng mL^−1^ for dichlorvos [69].

In practical applications, researchers have combined processable polymeric substrates, such as fibers, foams, and films, with MOF materials to form composite materials, which could enhance their stability and functionality and overcome the brittleness of pure MOF materials. Ma’s group introduced zirconium-based MOFs (Zr-MOFs) with Lewis acid catalytic sites into fibrous bacterial cellulose (BC) substrates to synthesize MOF nanozyme aerogel composite materials. The high-activity Zr-MOF nanozyme, combined with the flexible and macroporous BC substrate, provides more catalytic sites, enabling rapid hydrolysis of organophosphorus pesticides or neurotoxins. They studied the continuous hydrolysis performance of dichlorvos, using a micro-pump to inject the aqueous solution of the model compound at a rate of 0.1 mL min^−1^, performing spectral analysis on every 1 mL of filtrate to measure the conversion rate of DMNP. The half-life of hydrolysis was 1 min. Within the first 11 mL, dichlorvos was quantitatively converted into the non-toxic product dichlorvos, and after the 13th mL, the conversion rate of dichlorvos slightly decreased to 94% [70]. Zhang et al. [71] created defect structures in MOF materials to increase active sites. They doped L-cysteine (L-Cys) into the cobalt-based zeolitic imidazolate framework (ZIF-Co) to synthesize ZIF-Co-Cys nanozymes with high oxidase-like activity. This method enabled fluorescence and photothermal dual-mode detection of dichlorvos within the ranges of 2~100 ng mL^−1^ and 10~10,000 ng mL^−1^, with detection limits of 1.64 ng mL^−1^ and 0.084 ng mL^−1^, respectively [71]. Cai’s group reported a novel method of creating defects using mixed-linker MOFs, resulting in MIL-OH-D having a higher peroxidase-like activity compared to the original material. The detection range for dichlorvos was 5~300 ng mL^−1^, with a detection limit of 2.06 ng mL^−1^ [72]. Moreover, Yu’s group attached UiO-66-NH_2_ materials to MF sponges using polydopamine (PDA) and then modified them with dodecylbiguanide hydrochloride (DDT) for hydrophobicity. This effectively facilitated the catalytic degradation of organophosphorus pesticides. After 70 min, the hydrolysis rate of parathion by DDT-UiO-66-NH_2_@MF reached 66.6% [73].

Regulating metal nodes or ligand structures is a commonly employed strategy to enhance catalytic activity. Chai’s group introduced additional active centers by synthesizing a dual-site porphyrin MOF nanozyme, which enhances nanozyme activity through charge density distribution interactions. Both the V node and FeTCPP ligand exhibit catalytic activity, with FeTCPP playing a dominant role. They first successfully prepared a 2D Fe-porphyrin MOF derived from MXene, named VTCPP(Fe). Based on this MOF nanozyme, it exhibited peroxidase-like and catalase-like activities under acidic and neutral/alkaline conditions, respectively. Furthermore, luminol-H_2_O_2_ catalyzed by V-TCPP(Fe) showed enhanced chemiluminescence (CL) behavior. They then create a hybrid V-TCPP(Fe)/AChE@ZIF-8 system capable of dual-mode detection of organophosphorus pesticides. The absorbance/CL intensity significantly increased as the organophosphorus pesticides concentration increased. The detection limits for colorimetric and fluorescent detection of chlorpyrifos were 0.61 nM and 0.13 nM, respectively [74]. Liu et al. [75] used Cu^2+^ as the metal node and 2-aminoisophthalic acid (NH_2_-BDC) as the organic linker to prepare a bifunctional MOF nanozyme, NH_2_-CuBDC. By using this MOF multifunctional nanozyme as the recognition element, they established a dual-mode sensor that combines colorimetric and fluorescent detection, leveraging its peroxidase-like activity and fluorescence properties at 448 nm. Using chlorpyrifos as the organophosphorus pesticide model, the detection limits for chlorpyrifos in colorimetric and fluorescent modes were 1.57 ng mL^−1^ and 2.33 ng mL^−1^, respectively. The blocking signals in colorimetric and fluorescent detection increased proportionally with chlorpyrifos concentration within the ranges of 1.8~180 ng mL^−1^ and 4.5~450 ng mL^−1^, respectively [75]. Some studies proposed combining metal ions in MOF materials with aggregation-induced emission agents (AIEgens) to develop nanozymes with both phosphatase activity and fluorescence properties. They combined Zr^4+^ with 1,1,2,2-tetrakis(4-carboxyphenyl)ethylene (TCPE) to form Zr-TCPE MOF nanozyme materials. These materials enable colorimetric/fluorescent dual-mode detection of parathion within the ranges of 1.82~181.69 μM for colorimetric mode, with a detection limit of 0.178 μM, and 0.36~181.69 μM for fluorescent mode, with a detection limit of 0.195 μM [76]. Recent studies have found that two-dimensional (2D) MOF materials exhibit more exposed active sites and easier access to these sites, promoting enhanced catalytic activity. For example, Yang et al. [77] successfully synthesized ultrasmall AuNPs/2D MOF hybrid nanosheets (UsAuNPs/2D MOF). The synergistic interaction between AuNPs and 2D MOF nanosheets significantly enhanced the peroxidase-like activity. Based on this, they established a biosensor for colorimetric detection of organophosphorus pesticides, capable of rapidly detecting dichlorvos. The linear range was 1.7~42.4 μM with a detection limit of 1.7 μM. Within this range, the weakening of the degree of blueness in the UsAuNPs/2D MOF hybrid reaction system could be visually observed [77].

### 4.4. Single-Atom Nanozymes (SAzymes)

Table 6 has listed the performance of several single-atom nanozymes for the degradation and detection of organophosphorus pesticides.

In recent years, single-atom nanozymes have garnered significant attention from researchers both domestically and internationally. These nanozymes combined cutting-edge single-atom technology with intrinsic enzyme-like active sites. SAzymes consist of isolated metal atoms well-dispersed on a carrier. The atomic dispersion of metal centers maximizes the atomic utilization efficiency and density of active sites, while porous carriers provide abundant channels for mass transfer. Single-atom nanozymes hold great potential as the next generation of nanozymes [92]. Emerging single-atom nanozymes, renowned for their superior stability and efficient catalytic activity, have been rapidly developed for designing biosensors. M-N-C (where M represents Fe, Cu, Co, etc.) nanozymes exhibit MNx sites similar to natural metal enzymes. For instance, X. Niu et al. [93] proposed a Fe-N-C single-atom nanozyme that demonstrates unprecedented peroxidase-like activity, with a specific activity of 57.76 U mg^−1^, comparable to natural horseradish peroxidase (HRP). Most research teams currently focus on studying the peroxidase-like activity of Fe-based nanomaterials. However, Y. Wu et al. [79] reported in 2019 that Fe-N-C SAzymes possess oxidase-like nanozyme activity. They used TMB as a substrate and combined it with AChE to develop a biosensor for detecting the content of organophosphorus pesticides. The detection range for parathion-ethyl was 0.1~10 μg mL^−1^, with a detection limit of 0.97 ng mL^−1^ [79]. Y. Zhang et al. [80] utilized a metal-complex pyrolysis method to synthesize Fe-N-C nanozymes and designed a colorimetric biosensor for organophosphorus pesticides detection. Acid phosphatase (ACP) hydrolyzes the substrate L-ascorbic acid-2-phosphate (AAP) to generate ascorbic acid (AA), which inhibits the oxidation of TMB, while organophosphorus pesticides inhibits the enzymatic activity of ACP, impeding AA production. The system’s blue color deepens gradually as the concentration of omethoate increases, and this sensor achieves a detection limit of 0.4177 nM for omethoate [80]. Y. Wu et al. [81] fabricated Cu-N-C nanozymes with high concentrations of isolated copper atoms on nitrogen-doped carbon nanosheets, exhibiting excellent peroxidase-like activity. Based on this, they combined it with natural AChE and ChOx to create a tri-enzyme cascade biosensor (ACC) for colorimetric organophosphorus pesticides detection. Under optimized conditions, this system showed high sensitivity and selectivity for organophosphorus pesticides detection, with a wide linear range of 1~300 ng mL^−1^ for parathion-ethyl as the organophosphorus pesticides model, and a detection limit of 0.60 ng mL^−1^ [81]. G. Song et al. [82] constructed a bioactive paper based on single-atom Ce-N-C nanozymes. As the concentration of single-atom Ce-N-C nanozymes increased, AChE activity was inhibited, leading to more TMB oxidation and a bluer color of the bioactive paper. The color of the bioactive paper is positively correlated with the level of pesticide residues, with detection limits of 55.83 ng mL^−1^ for omethoate and 71.51 ng mL^−1^ for methamidophos, and a detection range of 100~700 μg mL^−1^, achieving a recovery rate of 84.09~104.68% [82]. Q. Chang et al. [83] anchored Ce atoms symmetrically on a porous N-doped carbon carrier to form single-atom nanozymes (CeN_4_-SAzyme) with specific peroxidase-like activity. Based on this peroxidase-like activity, they combined this nanozyme with AChE and ChOx to create a tri-enzyme cascade biosensor. This sensor achieved detection limits of 0.56 ng mL^−1^ and 0.67 ng mL^−1^ for dichlorvos and chlorpyrifos, respectively, in the range of 1 ng mL^−1^ to 1 μg mL^−1^ and demonstrated robust interference resistance [83]. Z. Zhao et al. [84] synthesized Fe-N-C nanozymes with atomically dispersed dual Fe centers on N-doped porous carbon. Due to the predominant role of reactive oxygen species (•O^2−^) in catalytic reactions, these nanozymes exhibit excellent oxidase-like activity. The addition of organophosphorus pesticides causes a decrease in thiocholine (TCh) concentration, and the solution’s color changes from light blue to red under UV irradiation. A fluorescence ratio assay was designed, with the fluorescence ratio increasing linearly from 0.005 to 50 ng mL^−1^ and a detection limit of 1.9 pg mL^−1^ [84]. Qin’s group analyzed the oxygen reduction characteristics and peroxidase-like activity of Fe single-atom nanozymes (Fe SACs), achieving sensitive photoelectrochemical (PEC) analysis. The atomically dispersed FeN_4_ sites in Fe SACs are effective active components that accelerate the sluggish oxygen reduction reaction, reducing interfacial charge recombination and enhancing PEC performance. By loading Fe SACs on the surface of Cu_2_O/Ti_3_C_2_T_X_, they successfully synthesized Fe SACs/Cu_2_O/Ti_3_C_2_T_X_ nanozymes and constructed a robust PEC sensing platform for sensitive detection of acetylcholinesterase activity and organophosphorus pesticides. This platform provided a wide linear response range of 0.5~600 ng mL^−1^ for paraoxon-ethyl, with a detection limit of 0.08 ng mL^−1^ [85].

Recent studies have revealed that M-N-C SAzymes primarily exhibit favorable peroxidase-like activity in acidic environments, with little attention paid to alkaline environments [94,95]. Therefore, developing M-N-C SAzymes with high POD-like activity in alkaline environments is of significant importance. Z. Luo et al. [86] synthesized a series of M-N-C SAzymes (M=Fe, Co, etc.) and anchored them onto nitrogen-doped carbon nanotube aerogels. Experiments showed that Co-N-C exhibited the highest catalytic activity in alkaline media. Based on the specific inhibition of AChE by impurities in organophosphorus pesticides, a chemiluminescence biosensor based on Co-N-C was further developed for organophosphorus pesticides detection, with a detection range of 0.8 ng mL^−1^~500 ng mL^−1^ and a detection limit of 0.37 ng mL^−1^ [86]. Zhong et al. [87] investigated a colorimetric sensor array that combines Fe-N-C nanozymes and Cu-N-C nanozymes with natural AChE. This array can perform fingerprint analysis (LDA) for eight organophosphorus pesticides: isopropyl parathion, diazinon, crotoxyphos, ethyl bromophos, dichlorvos, chlorpyrifos, ethion, and omethoate. The analysis demonstrated that all eight organophosphorus pesticides at a concentration of 1 ng mL^−1^ could be successfully identified, with different concentrations of organophosphorus pesticides appearing independently in fixed regions of LDA. This indicates the sensor array’s strong discrimination capability for different organophosphorus pesticides. Within the linear range of 20 ng mL^−1^ to 100 ng mL^−1^, the detection limits for Dichlorvos, Ethion, and Omethoate were 1.04 ng mL^−1^, 1.24 ng mL^−1^, and 0.78 ng mL^−1^, respectively.

High-temperature pyrolysis and doping with transition metal elements can reveal the active sites of MNx supported on porous carbon carriers, significantly enhancing catalytic activity. Scholars have developed a colorimetric aptamer sensor by using Fe-Co magnetic nanoparticles (Fe-Co MNPs) and Fe-N-C nanozymes based on pyrolysis reactions. Fe-N-C nanozymes and Fe-Co MNPs were synthesized through high-temperature pyrolysis of Fe-ZIF-8 and Fe-ZIF-67, respectively. After amino functionalization, they were modified with carboxylated aptamers and cDNA to form aptamer sensors for the detection of organophosphorus pesticides. The peroxidase-like activity of Fe-N-C nanozymes catalyzes the TMB-H_2_O_2_ color system, turning the solution blue–green. The detection limits for four pesticides—phorate, profenofos, isocarbophos, and omethoate—are as low as 0.16 ng/mL, 0.16 ng/mL, 0.03 ng/mL, and 1.6 ng/mL, with linear ranges of 0.5~5000 ng/mL, 0.5~5000 ng/mL, 0.1~5000 ng/mL, and 5~5000 ng/mL, respectively. This method shows excellent specificity, providing an effective means for detecting residual organophosphorus pesticides in vegetables [88]. Moreover, scholars have doped ZIF-8 with Fe atoms, synthesized single-atom iron nanozymes with peroxidase-like activity through high-temperature pyrolysis, and then functionalized them with APTES-GA aldehyde-modified nanozymes. The resulting aptamer sensor, coupled with amino-modified aptamers, exhibited a broader colorimetric detection range of 10^−12^ to 10^−2^ M for three organophosphorus pesticides: ethyl parathion, dichlorvos, and omethoate, with detection limits of 60.97 fM, 13.62 fM, and 7.54 fM, respectively [89]. In previous studies, Wang et al. [90] reported a method for preparing Fe single-atom nanozymes (Fe-N-C) using alkali lignin, dicyandiamide, and Fe^3+^ as carbon carriers, nitrogen donors, and metal active centers, respectively. This method involves high-temperature pyrolysis of alkali lignin. The sensor, combined with AChE, was used to detect chlorpyrifos with a linear range of 0.05~10.0 μg mL^−1^ and a detection limit of 2.11 ng mL^−1^ [90].

Most nanozymes, including single-atom nanozymes, show enzyme-like activity, such as catalase and oxidase functions. Chen et al. [91] attempted to introduce axial N ligands to construct Fe-N_5_ coordinated single-atom nanozymes, endowing them with significant oxidase-like activity. Using ZnCl_2_ and SiO_2_ as dual template agents and Fe^2+^ as the iron source, they constructed a polymerization–pyrolysis–evaporation–etching (PPEE) strategy to synthesize single-atom nanozymes with FeN_5_-active centers, designated Fe SAs/N_5_-pC-4. Leveraging the excellent oxidase-like activity of this nanozyme, they successfully constructed an AChE/ATChI/Fe SAs/N_5_-pC-4 biosensor for colorimetric analysis of organophosphorus pesticides. The sensor exhibits a good linear relationship within the concentration range of 0.001 to 20 μg mL^−1^, with a detection limit as low as 0.0006 μg mL^−1^ [91].

### 4.5. Carbon-Based Nanozymes

What Table 7 shows is the application and performance of carbon-based nanozymes for the degradation and detection of organophosphorus pesticides.

Carbon-based nanozymes, as metal-free catalysts, exhibit excellent electron transfer capabilities. Their functional groups, including carbonyl (−C=O), carboxyl (−COOH), and hydroxyl (−OH), serve as catalytically active sites [101]. Numerous successful applications of carbon-based nanozymes in the specific detection of organophosphorus pesticides have been reported in current research.

#### 4.5.1. Carbon Nanosheets

Dang et al. [96] synthesized phosphorus–oxygen (P, O) dual-doped carbon nanosheets (POCNS) using a one-step pyrolysis method. The dual-doping accelerates electron transfer, enhancing the catalytic activity of the carbon nanozymes. The nanosheet structure increases the exposure of active sites. Leveraging the high peroxidase-like activity of POCNS, they designed a colorimetric detection platform for organophosphorus pesticides such as chlorpyrifos. The linear range for detecting chlorpyrifos was 1~200 μg L^−1^, with a detection limit of 0.31 μg L^−1^. This provides a straightforward approach for synthesizing carbon nanozymes with high peroxidase-like activity [96]. Kumar’s group modified carbon nanotubes with Cu, Ni, and Co, creating highly active Cu@CNT, Ni@CNTs, and Co@CNTs nanocomposites. The sensor array constructed from these materials showed excellent responses and recognition for eight organophosphorus pesticides: carbendazim (CBZ), isoproturon (ISP), profenofos (PFF), atrazine (ATZ), diethyl cyanophosphate (DCNP), deltamethrin (DTM), and diethyl phosphoramidate (DPM). The detection limits for CBZ, DTM, and ISP in the concentration range of 1~8 μM were 10.8 nM, 28.8 nM, and 16.8 nM, respectively [97].

#### 4.5.2. Graphene and Graphene Oxide

Graphene-based nanozymes demonstrate strong binding with organophosphorus pesticides through π–π stacking interactions, which inhibit their catalytic activity. Zhu et al. [98] combined three types of heteroatom-doped graphene nanozymes—nitrogen-doped graphene (NG), nitrogen–sulfur co-doped graphene (NSG), and graphene oxide (GO) in sensor arrays. The cross-response of these arrays generated sensor data, facilitating the differentiation of various analytes. This sensor array was capable of distinguishing five pesticides: lactofen, fluoroxypyr-meptyl, bensulfuron-methyl, fomesafen, and diafenthiuron. Linear discriminant analysis (LDA) generated two-dimensional score plots for different pesticides ranging from 5 to 500 μM. Additionally, the sensor array demonstrated excellent performance, distinguishing between 11 datasets across a concentration range of 0~1000 μM. Chu et al. [99] synthesized oxidized graphene (GO) rich in C=O functional groups and found that it exhibited excellent peroxidase-like activity. Utilizing the peroxidase-like activity of GO and combining it with AChE and ChOx, they designed a colorimetric sensor for detecting organophosphorus pesticides at nanomolar levels. The detection limits for omethoate, methyl parathion, and chlorpyrifos were estimated at 2, 1, and 2 ng mL^−1^, respectively, with linear ranges of 2~200, 1~50, and 2~100 ng mL^−1^, respectively.

#### 4.5.3. Carbon Dots (CDs)

CDs are highly regarded as carbon nanomaterials because of their low cost, non-toxic nature, facile surface functionalization, high water solubility, excellent biocompatibility, and remarkable photostability. CDs typically contain functional groups such as nitrogen or carboxyl groups, making them ideal candidates for constructing aerogels. Yi et al. [100] synthesized a three-dimensional porous copper–carbon dot aerogel (Cu-CDs) using a copper-ion-induced self-assembly method. They coupled Cu-CDs with AChE and ChOx to create a colorimetric biosensor based on multi-enzyme cascade catalysis. Selecting dichlorvos as the organophosphorus pesticide model, the linear range was 0.02~0.3 μM, with a detection limit as low as 7.6 nM.

### 4.6. COF-Based Nanozymes

From Table 8, the performance of several COF-based nanozymes in the degradation and detection of organophosphorus pesticides is clearly visible.

In recent years, COFs as a novel class of porous materials, have garnered significant attention due to their high thermal stability [107,108], permanent and adjustable porosity [109], low density [110], and large surface area. Leveraging the nanozyme activity of COFs and applying them to the detection of organophosphorus pesticides has become a viable strategy.

By utilizing catalytically active COF shells as carriers to encapsulate another nanozyme, overall dispersion and catalytic activity can be enhanced. Building on this concept, Li et al. [102] synthesized a novel core–shell nanocomposite, Prussian blue@Fe-covalent organic framework@Au (PB@Fe-COF@Au). The triple peroxidase-like activity sources of this composite nanozyme include Prussian blue nanoparticles (PBNPs) containing coexisting Fe^2+^ and Fe^3+^, metalloporphyrins, and Au nanoparticles (AuNPs). Based on its prominent peroxidase-like activity, a colorimetric detection method combining AChE and organophosphorus pesticides was proposed. PB@Fe-COF@Au exhibited a response to AChE that was far superior to PBNPs and PB@Fe-COF. Within the concentration range of 10~800 ng mL^–1^, the detection limit for dichlorvos was as low as 0.17 mU mL^–1^. Additionally, to cater the practical pesticide detection scenarios, a spherical hydrogel device based on PB@Fe-COF@Au was designed, enabling rapid and convenient detection of dichlorvos through visual observation of the device’s blue hue [102]. Xiao et al. [103] constructed a novel D-A structured COF (DAFB-DCTP COF) with π-conjugated skeletons. Upon interaction with the organophosphorus toxin diethyl cyanophosphate (DCNP), it formed a pyridine-phosphorylated complex, which blocks the catalytic sites of the nanozyme and inhibits its photosensitive properties, thereby inducing a colorimetric reaction by suppressing the generation of p-NP. Within the range of 0~1.308 mM, there is a good linear relationship between absorbance and DCNP concentration, achieving a detection limit of 16.8 μM [103]. Wen’s group encapsulated methylene blue (MB) with a high loading capacity and manganese oxide (MnO_2_) with oxidase-like activity within COF materials to synthesize a novel nanozyme MB/COF@MnO_2_ (MCM). Based on this MCM composite probe, a fluorescence (FL)/electrochemical (EC) dual-ratiometric sensing strategy was designed to detect organophosphorus pesticides. Using dichlorvos as the organophosphorus pesticides model, the fluorescence response detection limit within the concentration range of 1~200 ng mL^–1^ was 0.083 ng mL^–1^, and the electrochemical response detection limit within the range of 0.25~80 ng mL^–1^ was 0.026 ng mL^–1^ [104]. Integrating light-responsive oxidase mimics into point-of-care testing (POCT) has emerged as a more environmentally friendly and cleaner approach. Liang et al. [105] reported a POCT-sensing platform triggered by a smartphone flashlight to activate the nanozyme activity of COFs. This platform can detect dichlorvos within the concentration range of 8~2000 ng mL^–1^, with a linear relationship and a detection limit of 1.29 ng mL^–1^ [105]. Furthermore, Zhang’s group introduced valine into a covalent organic framework nanozyme TAPB-DMTP-COF (TAPB: 1,3,5-tris (4-aminophenyl) benzene; DMTP: 2,5-dimethoxyterephthaldehyde). By leveraging the amino group in valine, they successfully introduced CeO_2_, ultimately forming the COF-OMe@Valine-CeO_2_ composite. The synergistic effect of Ce (IV)/Ce (III) effectively polarizes and hydrolyzes the P=O bond in methyl paraoxon, and the nanocomposite also adsorbs methyl paraoxon through π–π stacking interactions. An electrochemical sensing platform constructed using this composite achieved a low detection limit of 0.011 μmol L^–1^ within the linear range of 0.034~76 μmol L^–1^ [106].

## 5. Conclusions and Prospects

In recent years, the issue of organic phosphorus pesticides residues has garnered significant public attention, and intensifying research on their degradation and detection is of profound practical significance. Modern organic phosphorus pesticides face numerous challenges, including extensive usage, complex compositions, high toxicity, significant environmental risks, difficulty in detection, and ease of migration and transformation within the environment. Consequently, several research teams have focused on addressing these issues. Among these approaches, utilizing nanozymes for the detoxification and detection of organophosphorus pesticides is a more reliable, sensitive, rapid, and practical strategy. Nanozymes, as a novel class of artificial enzymes, integrate the properties of nanomaterials and enzymes, which hold broad prospects for application in the field of environmental safety, particularly for multifunctional degradation and detection of organophosphorus pesticides. This review discusses the characteristics, hazards, and migration pathways of organophosphorus pesticides in the environment and comprehensively summarizes the types of nanozymes used for their degradation and detection, along with their respective degradation and detection performances.

Looking forward, the discovery and application of high-efficiency, multifunctional nanozymes remain promising. Several avenues warrant further exploration:

1. Development of dual-function nanozymes: Efforts should be focused on creating nanozymes that integrate both degradation and detection functionalities, aiming to achieve integrated removal and real-time monitoring. Such approaches would advance the development of innovative functional environmental materials.
2. Evaluation of long-term environmental and health impact: assessing and monitoring the long-term effects by using nanozymes on the environment and within the human body is essential to ensure their environmental friendliness and health safety.
3. Manufacturing processes for industrial-scale production: investigating manufacturing processes suitably for large-scale industrial production and aligning them with practical applications in areas such as agricultural production, food processing, and environmental safety is crucial for widespread adoption.

In the future, the potential of nanozymes in mitigating the adverse effects of organophosphorus pesticides can be fully realized, which would contribute to a safer, healthier, and more sustainable environment.

## Figures and Tables

**Table 1:** The molecular structure and LD_50_ (mg/kg) of some common organophosphorus pesticides.

Compound	Molecular Structure	Organism	Route	LD_50_ (mg/kg)
Bromophos-ethyl	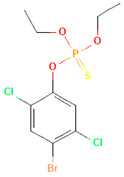	rat	oral	52
Chlorpyrifos	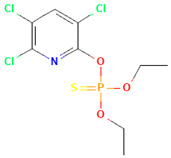	rat	skin	202
Famphur	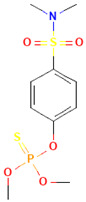	rat	skin	400
Parathion	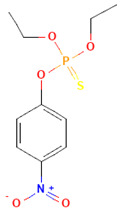	human	oral	3
Fenchlorphos	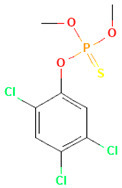	dog	oral	500
Sulfotep	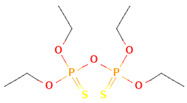	dog	oral/	5

Data sources in Table 1: https://pubchem.ncbi.nlm.nih.gov. (accessed on 4 December 2024)

**Table 2:** International distribution of organophosphorus pesticides.

Country	Water Body Name		Type of Organophosphorus Pesticides	Concentration (ng·L^−1^)	Reference
China	North China Plain		Summer	Winter	[16]
		Dimethoate	ND ^1^ ~ 23.11 ND ^1^ ~ 17.91 ND ^1^ ~ 16.23 ND ^1^ ~ 15.34	ND ^1^ ~ 16.23 ND ^1^ ~ 16.13 ND ^1^ ~ 15.47 ND ^1^ ~ 13.99	
		Dichlorvos			
		Methyl-parathion			
		Malathion			
	Yellow River	Dichlorvos	40.7		[17]
		Dimethoate	78.9		
		Omethoate	90.1		
	Haihe River	Dichlorvos	25.6		
		Dimethoate	70.8		
		Omethoate	42.8		
	Yangtze River	Dichlorvos	17.9		
		Dimethoate	17.5		
		Omethoate	ND ^1^~16.0		
	A certain river reservoir in South China	Methamidophos	20.95~35.90		[18]
		Dichlorvos	1.52~14.02		
		Acephate	22.42~436.9		
		Omethoate	10.70~55.09		
		Malathion	14.94~33.11		
		Chlorpyrifos	12.49~23.74		
		Quinalphos	10.49~20.21		
		Methamidophos	13.66~79.11		
		Triazophos	15.47~341.9		
	Jianghan Plain	Methamidophos	39.1		[19]
		Omethoate	48.3		
		Dimethoate	21.28		
		Diazinon	47.58		
Malaysia	Langat River	Quinalphos	17.8		[20]
		Chlorpyrifos	20.2		
		Diazinon	9.4		
		Chlorpyrifos	5057		[21]
USA	San Joaquin River	Diazinon	100		[22]
		Chlorpyrifos	35		
		Dimethoate	74		
Egypt	Nile River	Chlorpyrifos	580		[23]
		Triazophos	2600		
		Fenitrothion	1222		
		Triazophos	1488		

ND ^1^ = Not detected.

**Table 3:** Degradation and detection performance of metal-based nanozymes for organophosphorus pesticides.

Serial Number	Nanozyme	Target Compound	Linear Range	Detection Limit	Reference
1	Cu NPs		Fingerprints were used to distinguish organophosphorus pesticides at different concentrations (1, 5, 20, 50, 100 μg mL^−1^).		[33]
2	Bi_0.01_Au_1_	Paraoxon-ethyl	0.8~500 ng mL^−1^	0.41 ng mL^−1^	[34]
3	CoPcNS	Paraoxon	10~2000 μg L^−1^	1.1 μg L^−1^	[35]
4	Fe-PTs	Paraoxon	1~500 ng mL^−1^	0.28 ng mL^−1^	[36]
5	Fe/C/Bi_2_O_3_	Dichlorvos	10~100 μg L^−1^	0.6 μg L^−1^	[37]
6	Fe_3_O_4_@Au-Pt	Ethephon	0.1~500 μmol L^−1^	2.01 nmol L^−1^	[38]
7	Ir NPs	Malathion	0.1~5.0 μM	6 nM	[39]
8	Pd@PtBi_2_	Trichlorfon	0.1~100 ng mL^−1^	0.06 ng mL^−1^	[40]
9	AgNP	Chlorpyrifos	35~210 ppm	11.3 ppm	[41]
10	Pt-Ni NPs	Chlorpyrifos	colorimetric mode: 0.2~2.5 μg mL^−1^ photothermal mode: 0.005~3.0 μg mL^−1^	colorimetric mode: 1.2 ng mL^−1^ photothermal mode: 1.66 ng mL^−1^	[42]
11	PtPdNPs@g-C_3_N_4_	Trich	colorimetric mode: 0.28~50.0 ng mL^−1^; fluorescence mode: 0.11~50.00 ng mL^−1^	colorimetric mode: 0.083 ng mL^−1^; fluorescence mode: 0.033 ng mL^−1^	[43]

**Table 4:** Degradation and detection performance of metal oxide nanozymes for organophosphorus pesticides.

Serial Number	Nanozyme	Target Compound	Linear Range	Detection Limit	Degradation Property	Reference
1	CuO NPs	Malathion	0.1~5 mg L^−1^	0.08 mg L^−1^		[52]
2	GeO_2_ NPs	Paraoxon	0.1~50 pM	14 fM		[47]
3	Ag_2_O NPs	Fenitrothion, Chlorpyrifos, Omethoate, Triazophos, Methyl parathion, Trichlorfon		Identify organophosphorus pesticides at concentrations as low as 10 ng mL^−1^		[53]
4	γ-MnOOH NWs	Omethoate; Dichlorvos	5~50 ng mL^−1^; 1~10 ng mL^−1^	0.35 ng mL^−1^; 0.14 ng mL^−1^		[54]
5	MO@FHO	Malathion	PEC mode: 0.0001~0.5 μmol L^−1^; colorimetric mode: 0.001~50 μmol L^−1^	PEC mode: 0.017 ng mL^−1^; colorimetric mode: 0.8 nmol L^−1^		[55]
6	CeO_2_@NC	Paraoxon	3.0~100.0 μM		Rapid hydrolysis was achieved at low temperature (37 °C), low dosage (0.5 mg mL^−1^), and short time (10 min)	[56]
7	Au−pCeO_2_	Methyl parathion	5~200 μM	0.5 μM		[57]
8	CeO_2_@PDA@AuNCs-MIPs	Methyl parathion	0.45~125 nM	0.15 nM		[58]
9	In-CeO_2_	Dimethyl-p-nitrophenyl Phosphate			75% conversion rate after 6 h	[59]
10	Au@MnO_2-X_		0.01~50.0 ng mL^−1^	0.039 ng mL^−1^		[60]

**Table 5:** Degradation and detection performance of MOF-based nanozymes for organophosphorus pesticides.

Serial Number	Nanozyme	Target Compound	Linear Range	Detection Limit	Degradation Property	Reference
1	Ce-MOF	p-NPP			In CHES buffer (pH 9.0), the hydrolysis rate can reach 80% after 5 min of reaction.	[61]
2	Cu_4_Co_6_ ZIF	Pirimiphos-methyl	6 × 10^−4^~0.03 μM	0.151 nM		[62]
3	MIL-88B(V)	Ethion, Parathion, Dichlorvos and Paraoxon	0.055~10 μg mL^–1^, 0.04~10 μg mL^–1^, 0.06~10 μg mL^–1^, and 0.08~10 μg mL^–1^	0.018, 0.01, 0.02, and 0.027 μg mL^–1^		[63]
4	Mn/Fe-MIL (53)	Methyl parathion and Chlorpyrifos	10~120 nM; 5~50 nM	2.8 nM; 0.95 nM		[64]
5	Mn-ZIF-8	Chlorpyrifos	0.1~20 nM	54 pM		[65]
6	MIL-101(Fe)	Methyl parathion	8~800 ng mL^−1^	1 ng mL^−1^		[66]
7	Pt@ZIF-8@TMS	Malathion	0~500 ng mL^−1^	0.7 ng mL^−1^		[67]
8	MIL-888-NH_2_(Fe-MOF)	Dichlorvos	0.01~10.0 ng mL^−1^	2.9 pg mL^−1^		[68]
9	Ce/Zr-MOF@FP	Dichlorvos	0.5~500 ng mL^−1^	0.32 ng mL^−1^		[69]
10	Zr-MOF@BC	Dichlorvos			hydrolysis half-life: 1 min	[70]
12	ZIF-Co-Cys	Dichlorvos	fluorescence mode: 2~100 ng mL^−1^: photothermal mode: 10~10,000 ng mL^−1^	fluorescence mode: 1.64 ng mL^−1^; photothermal mode: 0.084 ng mL^−1^		[71]
13	MIL-OH-D	Dichlorvos	5~300 ng mL^−1^	2.06 ng mL^−1^		[72]
14	DDT-UiO-66-NH_2_@MF	Parathion			After 70 min, the hydrolysis rate reached 66.6%	[73]
15	VTCPP(Fe)	Chlorpyrifos		colorimetric mode: 0.61 nM; fluorescent modes: 0.13 nM		[74]
16	NH_2_-CuBDC MOF	Chlorpyrifos	colorimetric mode: 1.57 ng mL^−1^; fluorescent modes: 2.33 ng mL^−1^	colorimetric mode: 1.57 ng mL^–1^; fluorescent modes: 2.33 ng mL^–1^		[75]
17	Zr-TCPE MOF	Paraoxon	colorimetric mode: 1.82~181.69 μM; fluorescence mode: 0.36~181.69 μM	colorimetric mode: 0.178 μM; fluorescence mode: 0.195 μM		[76]
18	UsAuNPs/2D MOF	Dichlorvos	1.7~42.4 μM	1.7 μM		[77]

**Table 6:** Degradation and detection performance of single-atom nanozymes for organophosphorus pesticides.

Serial Number	Nanozyme	Target Compound	Linear Range	Detection Limit	Reference
1	Fe-N-C	Paraoxon-ethyl	0.1~10 μg mL^−1^	0.97 ng mL^−1^	[79]
2	Fe-N-C	O methoate	1~100 nM	0.4177 nM	[80]
3	Cu-N-C	Paraoxon-ethyl	1~300 ng mL^−1^	0.60 ng mL^−1^	[81]
4	Ce-N-C	Omethoate; Methamidophos	100~700 μg mL^−1^	55.83 ng mL^−1^; 71.51 ng mL^−1^	[82]
5	CeN_4_-SAzyme	Dichlorvos and Chlorpyrifos	1 ng mL^−1^~1 μg mL^−1^	0.56 ng mL^−1^; 0.67 ng mL^−1^	[83]
6	Fe_AC_/Fe_SA_-NC		0.005~50 ng mL^−1^	1.9 pg mL^−1^	[84]
7	Fe SACs/Cu_2_O/Ti_3_C_2_Tx	Paraoxon-ethyl	0.5~600 ng mL^−1^	0.08 ng/mL^−1^	[85]
8	Co-N-C		0.8 ng mL^−1^~500 ng mL^−1^	0.37 ng mL^−1^	[86]
9	Fe-N-C, Cu-N-C	Dichlorvos, Ethion, and Omethoate	20 ng mL^−1^~100 ng mL^−1^	1.04 ng mL^−1^, 1.24 ng mL^−1^; 0.78 ng mL^−1^	[87]
10	Fe-Co MNPs, Fe-N-C	Phorate, Profenofos, Isocarbophos, and Omethoate	0.5~5000 ng mL^−1^, 0.5~5000 ng mL^−1^, 0.1~5000 ng mL^−1^ and 5–5000 ng mL^−1^	0.16 ng mL^−1^, 0.16 ng mL^−1^, 0.03 ng mL^−1^ and 1.6 ng mL	[88]
11	Fe-N-C	Ethyl parathion, Dichlorvos, and O methoate	10^−12^~10^−2^ M	60.97 fM, 13.62 fM and 7.54 fM	[89]
12	Fe-N-C	Chlorpyrifos	0.05~10.0 μg mL^−1^	2.11 ng mL^−1^	[90]
13	Fe SAs/N_5_-pC-4		0.001~20 μg mL^−1^	0.0006 μg mL^−1^	[91]

**Table 7:** Degradation and detection performance of carbon-based nanozymes for organophosphorus pesticides.

Serial Number	Nanozyme	Target Compound	Linear Range	Detection Limit	Reference
1	POCNS	Chlorpyrifos	1~200 μg L^−1^	0.31 μg L^−1^	[96]
2	Cu/Ni/Co@CNTs	CBZ, DTM, ISP	1~8 μM	10.8 nM, 28.8 nM, 16.8 nM	[97]
3	NG, NSG, GO	Lactofen, Fluoroxypyr-meptyl, Bensulfuron-methyl, Fomesafen, and Diafenthiuron	5~500 μM		[98]
4	GO	Omethoate, Parathion methyl, and Chlorpyrifos	2~200, 1~50, 2~100 ng mL^−1^	2, 1, 2 ng mL^−1^	[99]
5	Cu-CDs	Dichlorvos	0.02~0.3 μM	7.6 nM	[100]

**Table 8:** Degradation and detection performance of COF-based nanozymes for organophosphorus pesticides.

Serial Number	Nanozyme	Target Compound	Linear Range	Detection Limit	Reference
1	PB@Fe-COF@Au	Dichlorvos	10~800 ng mL^–1^	0.17 mU mL^–1^	[102]
2	DAFB-DCTP COF	DCNP	0~1.308 mM	16.8 μM	[103]
3	MB/COF@MnO_2_	Dichlorvos	FL mode: 1~200 ng mL^–1^; EC mode: 0.25~80 ng mL^–1^	FL mode: 0.083 ng mL^–1^; EC mode: 0.026 ng mL^–1^	[104]
4	TpBTD COF	Trichlorfon	8~2000 ng mL^–1^	1.29 ng mL^–1^	[105]
5	COF-OMe@Valine-CeO_2_	Methyl paraoxon	0.034~76 μmol L^–1^	0.011 μmol L^–1^	[106]

## Data Availability

No new data were created for this review paper.
